# Physical and Psychological Factors Associated with Poor Self-Reported Health Status in Older Adults with Falls

**DOI:** 10.3390/ijerph17103548

**Published:** 2020-05-19

**Authors:** Jiyeon Kim, Mikyong Byun, Moonho Kim

**Affiliations:** 1 College of Nursing, Korea University, Anam-dong, Seongbuk-Gu, Seoul 02841, Korea; tortoi@korea.ac.kr (J.K.); mulanbb@korea.ac.kr (M.B.); 2 Department of Nursing Science, Catholic Kwandong University College of Medicine, 24 Beomil-ro 579beon-gil, Gangneung-si, Gangwon-do 25601, Korea; 3 Department of Hematology and Oncology, Gangneung Asan Hospital, University of Ulsan College of Medicine, 38 Bangdong-gil, Sacheon-myeon, Gangneung-si, Gangwon-do 25440, Korea

**Keywords:** fall, self-reported health status, older adults, public health

## Abstract

*Background:* Previous studies have proposed various physical tests for screening fall risk in older adults. However, older adults may have physical or cognitive impairments that make testing difficult. This study describes the differences in individual, physical, and psychological factors between adults in good and poor self-rated health statuses. Further, we identified the physical or psychological factors associated with self-rated health by controlling for individual variables. *Methods:* Data from a total of 1577 adults aged 65 years or over with a history of falls were analyzed, using the 2017 National Survey of Older Persons in South Korea. Self-reported health status was dichotomized as good versus poor using the 5-point Likert question: “poor” (very poor and poor) and “good” (fair, good, and very good). *Results:* Visual/hearing impairments, ADL/IADL restriction, poor nutrition, and depression were more frequently observed in the group with poor self-rated health. Multivariable logistic regression revealed that poor self-reported health was significantly associated with hearing impairments (OR: 1.51, 95% CI 1.12–2.03), ADL limitation (OR: 1.77, 95% CI 1.11–2.81), IADL limitation (OR: 2.27, 95% CI 1.68–3.06), poor nutrition (OR: 1.36, 95% CI 1.05–1.77), and depression (OR 3.77, 95% CI 2.81–5.06). *Conclusions:* Auditory impairment, ADL/IADL limitations, poor nutrition, and depression were significantly associated with poor self-reported health. A self-rated health assessment could be an alternative tool for older adults who are not able to perform physical tests.

## 1. Introduction

Falls in older adults can lead to serious injuries, including bone fractures and head trauma. Approximately 30% of adults aged 65 and over fall each year [1]. As the population ages, the socioeconomic burden related to falls increases. In the United States, the cost of falls was $38 billion in 2015 [2]. In South Korea, the estimated annual expenditure from falls in those aged 60 and over was 1.4 trillion KRW (Korean won), which is equivalent to USD 1 billion [3].

Assessing older adults at high risk for falls with objective indicators is critical to preventing falls, and several tests have been developed for this purpose [4,5,6]. However, many older people find it difficult to perform those physical assessment tests due to physical and cognitive impairments. Objective measurements require several physical actions, including one-leg standing, sit-to-stand, and stair ascent–descent tasks, which may be difficult for frail older adults to complete [7].

Subjective health statuses can suggest physical activity levels in older adults [8,9]. Older adults who recognize themselves as not being healthy are likely to have less muscle strength and be less active in everyday activities. Some researchers identified fall risk factors based on self-rated health status and reported an association between subjective health status and fall occurrence [10,11,12,13].

Research on how older adults who have experienced falls view their health status has been lacking. Additionally, the correlation between self-rated health and the physical and psychological factors associated with falls must be investigated in older adults who may not be able to perform physical tests. Analyzing the relationship between risk factors for falls in older adults who perceive themselves as non-healthy might be an efficient strategy for fall prevention.

According to the 2018 South Korea census data, 14.8 percent of the country is aged 65 and older [14]. The government of South Korea surveys the health status of these individuals every three years. This national data may provide insight into the association between fall-related factors and self-rated health in older adults who have fallen.

This study identifies the physical or psychological factors associated with the self-rated health status of older adults with a fall history.

## 2. Materials and Methods

### 2.1. Data Sources

Public data were gathered from the Health and Welfare Data Portal in Korea under the approval of the National Statistical Office. The data originated from the 2017 National Survey of Older Persons (NSOP) [15]. The database is made up of a stratified random sample of approximately 10,000 people in general housing facilities and is designed to represent the Korean older adult population. NSOP 2017 data were collected through in-person interviews, involving 10,299 seniors aged 65 or older (including 226 representatives) in 934 survey areas from June to August of 2017. A change in the questionnaire design had been approved by Statistics Korea (Authorization No.11771), based on pretests and expert review. The survey was conducted by 60 specialized surveyors (divided into 15 teams of four surveyors and with one supervisor each), who were trained by the research staff in advance.

### 2.2. Patient Selection and Study Design

**Figure 1:**
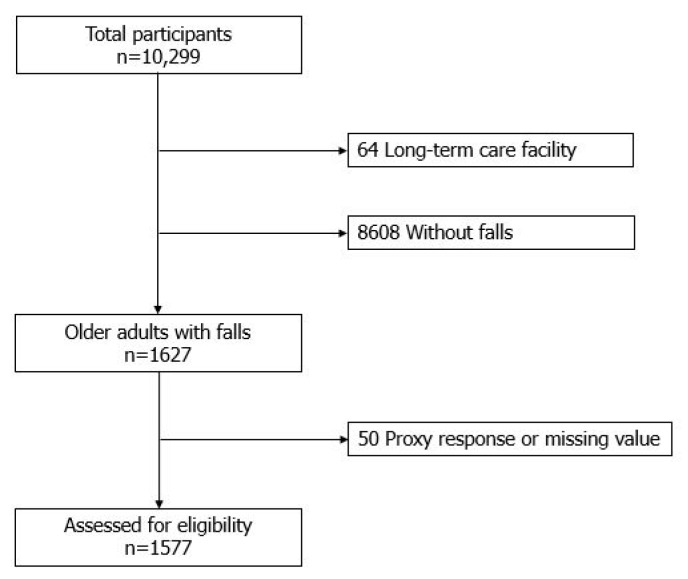
Consort diagram of study population inclusion.

A total of 1577 patients with a fall history were eligible from the original 10,299 participants (Figure 1). Patients with falls were those who responded affirmatively to the question “Have you experienced a fall within a year?”.

Using a descriptive and correlational study design, we first evaluated the predictors of subjective health assessments from three main categories (individual, physical, and psychological). We then adopted individual variable-adjusted models to find the physical and psychological factors that were more frequently associated with poor self-rated health status in a logistic regression analysis.

### 2.3. Ethical Considerations

Our Institutional Review Board (IRB) determined that this project was exempt from an IRB review because the research used existing national data and the information could not be linked to individual subjects (IRB No. 2020-01-004). Informed consent was not required as the data were de-identified and collected retrospectively.

### 2.4. Measurements

#### 2.4.1. Subjective Assessments of Health

Self-rated health was determined using the 5-point Likert question, “In general, how would you rate your health status?”. For this analysis, the five options were categorized into dichotomous variables: “poor” (very poor and poor) and “good” (fair, good, and very good).

#### 2.4.2. Individual Variables

The individual characteristics were classified into four subcategories: demographic, socio-economic, health status, and health-related behavior. First, the demographic variables included age, sex, marital status (living with or without a spouse), and living status (living alone, living with a spouse, or living with children). Second, the socioeconomic variables were the subject’s education level (0–6 years, 7–9 years, 10–12 years, or ≥13 years) and quartiles of household income (Q1 (lowest), Q2, Q3, Q4, or Q5 (highest)). Third, health status variables involved chronic diseases (hypertension, diabetes, dementia, or arthritis), body mass index (BMI), and the number of medications the individual was currently using (0, 1, 2, or ≥3). Finally, health-related behaviors consisted of exercise (none, <150 min a week, or ≥150 min a week), smoking (past/never or current), and drinking (none, ≤1 standard drink/day, or >1 standard drink/day).

Chronic disease was investigated using the question “Are you currently suffering from hypertension, diabetes, dementia, or arthritis for more than three months?” and “Have you been diagnosed by a doctor?”. Participants who answered “yes” to both questions were classified as having a chronic disease. An individual’s number of medications was based on their response to the question “How many physician-prescribed drugs have you been taking for the past three months or more?” Exercise for more than 150 min a week was considered to be within the recommended levels, in accordance with the World Health Organization (WHO) criteria [16]. Exercise levels were classified as within the recommended level, below the recommended level, and none. Those who responded “no” to the question “Do you usually exercise?” were classified as “none”. Drinking status was based on the National Institute on Alcohol Abuse and Alcoholism criteria [17]. In people aged 65 and over, drinking one standard drink (a 350 mL glass of beer) of alcohol per day is considered an appropriate intake in Korea. Intake of more than one standard drink of alcohol per day is considered excessive. Those who do not drink alcohol at all are classified as “none”.

#### 2.4.3. Physical Variables

Physical characteristics included visual impairment, hearing impairment, limited activities of daily living (ADL), and limited instrumental activities of daily living (IADL).

Sensory impairments were classified as either visual or auditory. Visual impairment was classified as “not impaired” in those who felt comfortable not wearing glasses or lenses, or using magnifying glasses, during daily activities. Those who said they were “uncomfortable” or “very uncomfortable” not using these aids were classified as “impaired”. Hearing impairment was classified as “not impaired” in individuals who were comfortable not wearing hearing aids in daily life. Those who responded that they were “uncomfortable” and “very uncomfortable” were classified as “impaired”.

Evaluation of ADL was based on the Korean Activity Daily Living scale, consisting of questions on seven categories: “dressing”, “face washing, brushing teeth, and shampooing”, “bathing”, “eating food”, “getting up and walking across the room”, “toilet use”, and “bowel and bladder control” [18]. The evaluation includes a three-point scale (total independence/partial dependence/total dependence), with total independence recorded as “no limitation” and partial and complete dependence recorded as “limitation”. Subjects who had ADL restrictions in more than one item were classified as having a “limitation of ADL”.

Limitations in IADL were determined using the Korean Instrumental Activity of Daily Living scale [18]. This consists of ten questions on categories including: “grooming”, “doing housework”, “preparing meals”, “washing clothes”, “picking up a set amount of medicine on time”, “managing money”, “going out to a nearby place”, “purchasing decisions, paying with money, and receiving change”, “making and receiving phone calls”, and “using transportation”. The evaluation also includes three- and four-point scales. The three-point scale (items 1–7) includes independence/partial dependence/total dependence, while the four-point scale (items 8–10) includes independence/little dependence/much dependence/cannot be done at all. Total independence was classified under “no limitation”, and partial, complete, little, much dependence, and cannot be done at all were recorded as a “limitation”. Subjects with restrictions in IADL in more than one item had a “limitation of IADL”.

Nutrition status was determined using the ‘Determine Your Nutritional Health’ questionnaire from the Nutrition Screening Initiative [19], which consists of ten questions with binary responses of “yes” or “no”. A “yes” response to each question is scored in the range of 1–4 and a “no” response is scored 0 points. The total score for 10 items is classified as 0–2: good nutrition, 3–5: moderate nutritional risk, and ≥6: high nutritional risk. Good nutrition scores were considered to be “good nutrition” and moderate and high nutritional risk were defined as “poor nutrition”.

#### 2.4.4. Psychological Variables

Depression was the primary psychological characteristic examined. Depression was determined using the Korean version of the 15-item Geriatric Depression Scale K (SGDS-K), which was proposed by Sheik and Yesavage and translated into Korean by Cho et al. [20]. Scores ranged from 0 to 15 on this scale. A previously published Korean study suggested that the optimal cut-off for SGDS-K scores during screening for major depressive disorders is ≥8; in this study, scores of ≥8 and <8 were classified as “depressed” and “not depressed”, respectively.

### 2.5. Data Analysis

**Figure 2:**
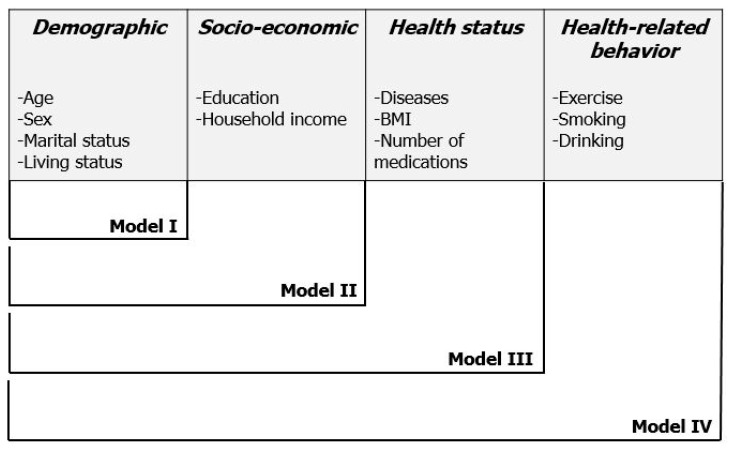
Suggested individual variable-adjusted models. The demographic, socio-economic, health status, and health-related behavior variables were combined in groups, and four models were established.

Descriptive statistics were performed. Differences in the subjective assessments of health status by individual, physical, and psychological variables were compared using the χ^2^ or *t*-test. Each independent variable was included in a univariate logistic regression analysis, and significant variables were chosen from this analysis for multivariate logistic regression analysis. We applied individual variable-adjusted models to control the possible confounding effects of individual factors. The individual variables, including demographic, socio-economic, health status, and health-related behavior subcategories, were combined in groups and entered into the logistic regression models (Model I: demographic only; Model II: demographic and socio-economic; Model III: demographic, socio-economic, and health status; Model IV: demographic, socio-economic, health status, and health-related behavior) (Figure 2). Odds ratios (ORs) and corresponding 95% confidence intervals (CIs) were also calculated. The level of statistical significance was set at less than 0.05. The data were analyzed using IBM SPSS statistical software, version 22.0 (IBM, Armonk, NY, USA).

## 3. Results

### 3.1. Incidence and Average Number of Falls

A total of 1577 patients (15.9%) experienced a fall within the past year, and there was an average of 2.1 falls within a year. The average number of falls was 2.5 in those in the poor self-rated health status group and 1.6 in the group with the good self-rated health status.

### 3.2. Subjective Assessments of Health

A total of 940 patients with falls (59.6%) reported that they were in poor health.

### 3.3. Differences in Individual Characteristics between Those with Good and Those with Poor Self-Reported Health Status

Differences in health status according to individual characteristics are summarized in Table 1. All demographic, socio-economic, health status, and health-related behavior characteristics (except BMI and smoking) were statistically significant between the good and poor self-reported health status groups.

### 3.4. Differences in Physical and Psychological Characteristics between Those with Good and Poor Self-Reported Health Statuses

There were significant differences between the physical characteristics of the two groups, including visual, hearing, ADL, IADL, and nutrition status (Table 2). Visual or hearing impairments, ADL or IADL restriction, and poor nutrition were more frequently observed in the group with a poor self-rated health status.

There were also significant differences between the psychological factors of the two groups, such as depression (Table 2). Most older adults in the group with good self-rated health status (84.8%) did not report depressive episodes.

### 3.5. Multivariable Logistic Regression Analysis of Factors Associated with Poor Self-Reported Health Status Using Individual Variable-Adjusted Models

To evaluate the physical and psychological factors affecting fall risk, we introduced four individual variable-adjusted models, as mentioned above. In Model IV, our study revealed that a poor subjective health status in older adults with a fall history was significantly associated with hearing impairment (OR: 1.51, 95% CI 1.12–2.03), ADL limitation (OR: 1.77, 95% CI 1.11–2.81), IADL limitation (OR: 2.27, 95% CI 1.68–3.06), poor nutrition (OR: 1.36, 95% CI 1.05–1.77), and depression (OR 3.77, 95% CI 2.81–5.06) on multivariate analysis (Table 3). Of note, visual impairment was associated with poor health status in Models I and II.

## 4. Discussion

The study hypothesized that older adults with a fall history are more likely to regard themselves as unhealthy and have more known fall-related risk characteristics. We identified statistically significant physical and psychological factors in older adults who have fallen and found that those who perceive their health as poor are more likely to have risk factors for falling.

Falls in older persons are a complex and multifactorial problem [21]. The aging process is accompanied by inevitable physical and psychological degeneration. Older adults are more vulnerable to both acute and chronic diseases and often take multiple medications. The physical changes of aging, combined with environmental risk factors, may increase fall risk. Thus, it is essential to develop efficient screening tools to prevent falls in older adults.

However, screening high-risk older patients for falls by physical testing is limited. Older persons may not be able to complete a physical assessment test due to weak muscle strength, joint pain, and cognitive or emotional comorbidities. In addition, physical examinations may be a financial burden to some older adults. 

Several trials have assessed cardiovascular disease (CVD) risk based on the subjective perception of health [22,23]. Duval et al. estimated CVD risk from the individual’s self-reported knowledge [22]. Their simple model showed that 95% of women and 87% of men were in concordance with the current risk model, which required blood pressure and serum cholesterol tests. Rantanel et al. recently reported that self-rated health has a positive association with physical health among people at risk for CVD [23].

Several current models assess fall risk [6]. However, these widely used tools were developed in different settings, including with hospitalized older patients or in community-dwelling older adults. In addition, the Berg Balance Scale and the Timed Up and Go Test require some dynamic physical activities [24,25]. The Downton Fall Risk Index, St. Thomas’s Risk Assessment Tool, and subjective risk rating of specific tasks (SRRST) do not involve physical movements. However, those tools require careful observation and measurement by healthcare providers [7,26,27]. Lastly, the Hendrich tool needs medical records or active drug ingredients to identify fall risk [28].

Previous studies have evaluated subjective health status and fall risk. Shimada et al. validated fall risk with the SRRST score, a tool that is based on day-center staff evaluating a person’s ability to walk, toilet, and go up and down stairs. A higher score indicates poor health status, which correlated with frail older adults with a fall history [7]. This tool was utilized, not by the older adults, but by skilled staff who observed them for a certain time. Knapik et al. studied the association between functional motor efficiency and the self-assessment of health in people aged 60 years old or older [9]. A 36-question survey evaluated the self-assessment of health. The results indicate that subjective health status correlated with physical fitness, particularly in women with chronic diseases. This may suggest that poor subjective health status could increase fall risk. Singh et al. analyzed the risk factors for falls in 3935 adults aged 55 and over by physical performance tests and self-rated health measures [10]. Falls were associated with diabetes, arthritis, urinary incontinence, poor self-rated health, and lower handgrip strength. Those who thought their health was equal to or less than fair (very poor/poor/fair/good/very good) had a higher fall risk. Lastly, Li et al. investigated the relationship between the confidence of self-management of falls (CSMoF) and fall risk self-perceptions [12]. They suggested that cognition-related factors (i.e., fear of falls and perceived limitations due to falls) should be emphasized in fall prevention programs.

This study demonstrates that subjective health awareness is a factor for assessing fall risk in older adults who cannot perform physical activity tests. For older adults who have difficulty with dynamic physical interventions and who are living in a community (except long-term care facilities), a health questionnaire may be a viable alternative to assessing fall risk.

We used large cohort data to investigate the association between physical and psychological factors and health recognition among older adults who have experienced a fall. Four models that controlled for variables enhanced accuracy. The analysis showed that visual impairment, ADL/IADL limitations, poor nutrition, and depression were statistically significant risk factors for falls, in line with previous research. Further study is required to more fully understand these associations.

There are some limitations to our study. First, the data analysis had a cross-sectional design, which could not find causality, but could only provide insight on the association. Second, there may be recall bias because the primary data source was interview responses. Third, we suggested that self-rated health could be a useful alternative to assessing fall risk in older adults who have already fallen. Further research is needed to validate our model in older adults who have not yet reported a fall.

Despite these limitations, our study was the first nationwide data analysis to describe the association between subjective health status and physical and psychological risk factors in older adults with a fall history using multi-step variable-adjusted models.

## 5. Conclusions

Auditory impairment, ADL/IADL limitations, nutritional problems, and depression were significantly associated with the subjective health-status recognition of older adults with a fall history. In addition, a self-rated health assessment or questionnaire could be an alternative tool for older patients who are not able to perform the physical tests that assess fall risk.

## Figures and Tables

**Table 1:** Differences in individual characteristics between those with good and poor self-reported health statuses (n = 1577).

Variables		Classification	Good	Poor	χ^2^	*p*
			(n = 637)	(n = 940)		
			n (%) or	n (%) or		
			M ± SD *	M ± SD *		
Demographic	Age		75.0 ± 6.3	76.2 ± 6.2	1191.31	<0.001
	Sex	Male	208 (32.7)	233 (24.8)	11.66	0.001
		Female	429 (67.3)	707 (75.2)		
	Marital status	Living with spouse	347 (54.5)	433 (46.1)	10.74	0.001
		Living without spouse	290 (45.5)	507 (53.9)		
	Living status	Alone	193 (30.3)	337 (35.8)	12.14	0.007
		Living with spouse	285 (44.7)	341 (36.3)		
		Living with children	138 (21.7)	234 (24.9)		
		Other	21 (3.3)	28 (3.0)		
Socio-economic	Education	0–6 years	410 (64.4)	687 (73.1)	32.95	<0.001
		7–9 years	81 (12.7)	127 (13.5)		
		10–12 years	98 (15.4)	104 (11.1)		
		≥13 years	48 (7.5)	22 (2.3)		
	Quantiles of household income	Q1 (lowest)	140 (22.0)	312 (33.2)	25.09	<0.001
		Q2	132 (20.7)	182 (19.4)		
		Q3	139 (21.8)	180 (19.1)		
		Q4	107 (16.8)	134 (14.3)		
		Q5 (highest)	119 (18.7)	132 (14.0)		
Health status	Disease	Hypertension	368 (57.8)	673 (71.6)	32.35	<0.001
		Diabetes	109 (17.1)	328 (34.9)	59.94	<0.001
		Dementia	7 (1.1)	33 (3.5)	8.93	0.003
		Arthritis	208 (32.7)	517 (55.0)	76.34	<0.001
	BMI **	Underweight (<18.5)	27 (4.3)	55 (5.9)	6.39	0.094
		Normal (≥18.5, <25)	428 (67.1)	583 (62.0)		
		Overweight (≥25)	182 (28.6)	302 (32.1)		
	Number of medication(s)	0	128 (20.1)	29 (3.1)	258.00	<0.001
		1	79 (12.4)	29 (3.1)		
		2	104 (16.3)	64 (6.8)		
		≥3	326 (51.2)	818 (87.0)		
Health-related Behavior	Exercise	None	183 (28.7)	429 (45.6)	53.67	<0.001
		<150 min a week	144 (22.6)	206 (21.9)		
		≥150 min a week	310 (48.7)	305 (32.5)		
	Smoking	Past/Never	585 (91.8)	872 (92.8)	0.47	0.495
		Current	52 (8.2)	68 (7.2)		
	Drinking	None	455 (71.4)	794 (84.5)	40.36	<0.001
		≤1 standard drink/day	79 (12.4)	72 (7.6)		
		>1 standard drink/day	103 (16.2)	74 (7.9)		

* M ± SD, mean ± standard deviation. ** BMI, body mass index.

**Table 2:** Differences in physical and psychological characteristics between those with good and those with poor self-reported health statuses (n = 1577).

Physical & Psychological Variables		Classification	Good	Poor	χ^2^	*p*
			(n = 637)	(n = 940)		
			n (%)	n (%)		
Physical	Visual impairment	No	410 (64.4)	488 (51.9)	24.00	<0.001
		Yes	227 (35.6)	452 (48.1)		
	Hearing impairment	No	524 (82.3)	656 (69.8)	31.36	<0.001
		Yes	113 (17.7)	284 (30.2)		
	ADL * limitation	No	604 (94.8)	738 (78.5)	79.64	<0.001
		Yes	33 (5.2)	202 (21.5)		
	IADL ** limitation	No	502 (78.8)	445 (47.3)	156.71	<0.001
		Yes	135 (21.2)	495 (52.7)		
	Nutrition	Good	369 (57.9)	284 (30.2)	120.21	<0.001
		Poor	268 (42.1)	656 (69.8)		
Psychological	Depression	No	540 (84.8)	473 (50.3)	196.19	<0.001
		Yes	97 (15.2)	467 (49.7)		

* ADL: Activities of daily living. ** IADL: Instrumental activities of daily living.

**Table 3:** Multivariable logistic regression analysis of factors associated with poor self-reported health status.

Variables	Model I	Model II	Model III	Model IV
	OR (95% CI)	OR (95% CI)	OR (95% CI)	OR (95% CI)
Visual impairment	1.31 (1.03–1.65) *	1.30 (1.03–1.65) *	1.26 (0.98–1.63)	1.26 (0.99–3.42)
Hearing impairment	1.51 (1.13–2.02) *	1.50 (1.12–2.00) *	1.51 (1.11–2.05) *	1.51 (1.12–2.03) *
ADL limitation	1.94 (1.256–3.00) *	1.95 (1.28–3.05) *	1.84 (1.16–2.91) *	1.77 (1.11–2.81) *
IADL limitation	2.66 (2.01–3.52) *	2.62 (1.98–3.47) *	2.34 (1.74–3.15) *	2.27 (1.68–3.06) *
Poor nutrition	1.89 (1.49–2.39) *	1.87 (1.47–2.37) *	1.36 (1.05–1.76) *	1.36 (1.05–1.77) *
Depression	3.84 (2.94–5.02) *	3.68 (2.81–4.83) *	3.87 (2.89–5.18) *	3.77 (2.81–5.06) *

Model I: adjusted for demographic (age, sex, marital, and living status) characteristics only. Model II: adjusted for demographic and socioeconomic (education and household income) characteristics. Model III: adjusted for demographic, socioeconomic, and health status (disease, BMI, and number of medications) characteristics. Model IV: adjusted for demographic, socioeconomic, health status, and health-related behavior (exercise, smoking, and drinking) characteristics. * *p* < 0.05.
